# Perforated Meckel's Diverticulitis in a Patient with Unknown Intestinal Malrotation: Clinical Pitfall

**DOI:** 10.1155/2021/5595803

**Published:** 2021-03-05

**Authors:** Marie Burgard, Floryn Cherbanyk, François Pugin, Bernhard Egger

**Affiliations:** Department of Surgery, HFR Fribourg-Cantonal Hospital Fribourg, Switzerland

## Abstract

Symptomatic Meckel's diverticulum is rare in adults. The most frequent complications are intestinal obstruction and diverticulitis. Diagnosis of Meckel's diverticulitis can be challenging due to nonspecific clinical manifestation of pain in the right lower abdominal quadrant, mimicking acute appendicitis. If associated with congenital malformation, such as intestinal malrotation, the anomalous anatomy makes the diagnosis even more challenging. In such cases, radiological imaging is essential to guide further management. We present a case of Meckel's diverticulitis in which physicians were initially misguided because of the atypical clinical presentation. Yet, anamnestic details directed to a potential underlying malformation, leading to supplementary radiological examination and the final diagnosis.

## 1. Case Report

Meckel's diverticulum (MD) mostly remains asymptomatic with a lifetime onset of complications of 4% but decreasing with age [1]. Therefore, symptomatic MD in adults is rare. The most common complications are intestinal obstruction and diverticulitis, which may lead to perforation, and gastrointestinal bleeding. Diverticulitis of such a diverticulum usually presents as acute right or diffuse abdominal pain with or without localized peritonitis. Preoperative diagnosis is achieved in only 5-15% of cases, and inflamed MD is often misdiagnosed as acute appendicitis [2]. Localized left-sided abdominal pain as a manifestation of Meckel's diverticulitis is highly unusual but can occur in patients with intestinal malrotation.

Acute diverticulitis of such a diverticulum may lead to perforation, and treatment is emergency surgery. Emergency physicians should be aware of this pathology and keep it in mind as a differential diagnosis of acute abdominal pain. Our case illustrates Meckel's diverticulitis in a patient presenting with left abdominal pain due to an undiagnosed intestinal malrotation.

A 65-year-old male patient with a medical history of sigmoid diverticulosis presented to his general practitioner with mild acute abdominal pain localized in the left lower quadrant. The clinical examination showed no abdominal tenderness, and the patient had normal blood values. The patient was discharged with a diagnosis of mild sigmoid diverticulitis and treated with oral antibiotics. Twenty-four hours later, he presented to our emergency department with increasing abdominal pain, fever of 38.1°C, and nausea. A detailed history revealed possible malrotation of the intestine. Clinical examination revealed localized periumbilical and left lower quadrant abdominal tenderness. Laboratory tests demonstrated a normal white blood cell count but increased C-reactive protein (52 mg/l).

**Figure 1:**
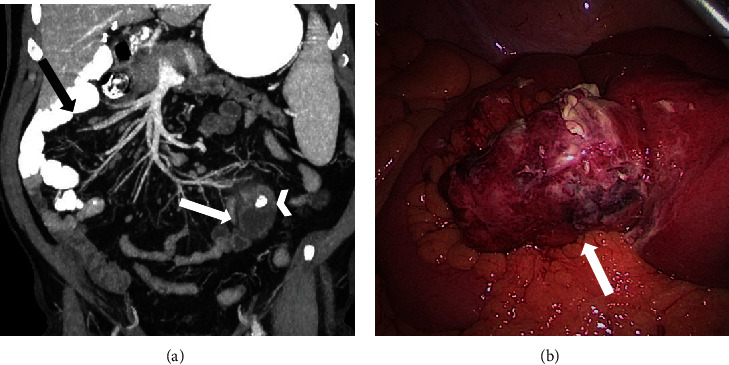
(a) Coronal reformatted contrast-enhanced CT images thickened with maximal intensity projection demonstrate malrotation of the small intestine with the jejunum and a large part of the ileum placed on the right side of the abdomen (black arrow). In the left hemiabdomen is a blind-ending tubular structure arising from the distal small bowel (white arrow), which has a markedly thickened wall with extensive surrounding fat stranding. Note the intraluminal calcified lesion (white arrowhead) contained in the saccular structure. (b) Laparoscopic view of perforated Meckel diverticulitis.

**Figure 2:**
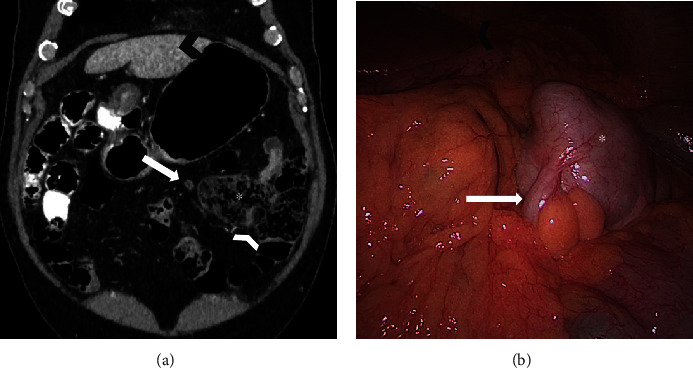
(a) Coronal reformatted contrast-enhanced CT image and (b) laparoscopic view demonstrate malrotation of the bowel with the ileocaecal valve (white arrowhead), the caecum (asterisk), and the normal appendix (white arrow) placed on the left side of the abdomen. Left hepatic lobe: black arrowhead.

Based on the clinical examination, the main diagnostic hypothesis was sigmoid diverticulitis. Nevertheless, the possibility of a malrotated intestine led to the differential diagnosis of acute appendicitis. Abdominal CT revealed a complete intestinal malrotation and inflamed diverticulum containing a calculus in the left upper quadrant without radiological signs of acute appendicitis (Figure 1(a)). The appendix was localized in the left lower quadrant (Figure 2(a)).

**Figure 3:**
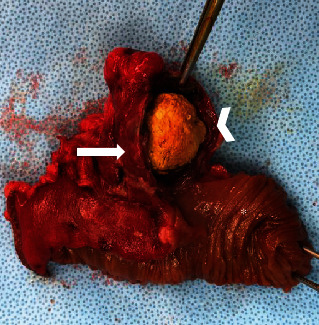
Surgical specimen: intestinal segment (asterisk), Meckel diverticulum (white arrow), and intradiverticular calculus (white arrowhead).

The patient underwent exploratory laparoscopy, showing a perforated diverticulum at the antimesenteric border of the ileum 50 cm from the ileocecal valve with local peritonitis (Figure 1(b)). A segmental intestinal resection with anastomosis and occasional appendectomy was performed. Perioperative microbiological examination showed polymicrobial intestinal flora. Examination of the 5 × 4.5 cm diverticulum confirmed the intradiverticular voluminous calculus (Figure 3). Definitive histology demonstrated inflamed gastric mucosa in the diverticulum, confirming the suspected diagnosis of a perforated MD. Due to the intestinal malrotation, the MD and appendix were localized in the left upper quadrant (Figure 2(b)). The postoperative outcome was uneventful, and the patient was discharged home on postoperative day 5 with oral antibiotics for another week. The 6-week outpatient control was favorable.

MD is the most common malformation of the gastrointestinal tract and is found in 0.14-0.4% of autopsies [3]. It is a remnant of the vitelline duct and, in adults, is usually located up to 100 cm from the ileocecal valve at the antimesenteric border of the ileum [4]. Anatomically, it is a true diverticulum with all layers of the gastrointestinal tract. Occasionally, it contains gastric or pancreatic mucosa [5].

Most MD cases stay asymptomatic with a lifetime complication rate of only 4% [1]. These complications most often occur before the age of 8 years; thus, symptomatic MD in adults is rare.

As the diverticulitis can lead to perforation, the treatment should be surgical. However, preoperative diagnosis can be difficult and is only achieved in 5-15% of cases, and inflamed MD is often misdiagnosed as acute appendicitis [2]. Abdominal CT can help make the diagnosis; yet, most MD cases are diagnosed intraoperatively during laparoscopy, especially if the diverticula do not contain a fecolith or foreign body. In patients with unknown congenital malformation, clinical diagnosis is even more difficult.

Intestinal malrotation is a congenital malformation due to the incomplete rotation of the fetal intestine around the superior mesenteric vessels. With an incidence of 0.2%, it is diagnosed during the first year of life in 90% of cases [6]. Incidental diagnosis in adult patients is rare. In patients with unknown intestinal malrotation, the diagnostic and therapeutic delay of acute abdominal pain can be important, with an increasing risk of morbidity and mortality. Physicians should keep in mind the differential diagnosis of acute appendicitis or Meckel's diverticulitis in a patient presenting with acute left abdominal pain, especially if there are anamnestic hints of intestinal malrotation. Abdominal CT may help make the diagnosis; if the CT scan is not conclusive, diagnostic laparoscopy may help and is mandatory in the presence of peritonitis.

Because of the possible diagnostic and therapeutic delay in patients with intestinal malrotation, occasional resection of noninflamed MD and the appendix should be considered when performing laparoscopy for other reasons.

## Additional Points

*Highlights.* (i) Meckel's diverticulum is often misdiagnosed as acute appendicitis. (ii) Intestinal malrotation can delay the diagnosis of abdominal pain. (iii) Appendicitis or Meckel's diverticulitis can be responsible for left abdominal pain.
